# Deaths under Anæsthetics

**Published:** 1908-02-15

**Authors:** 


### Deaths Under Anæsthetics.

We have on previous occasions drawn attention to the main factors involved in the various cases of deaths under chloroform or other anæsthesia. Lately, however, the public has once more, apparently, interested itself in the subject, and, to judge from the remarks which have appeared in the lay press, not to speak of the irresponsible expressions of opinion to be heard in the streets, there appears to be still a certain amount of doubt in the public mind as to how far, and in what way the doctor, as such, is responsible for these calamities. To a professional man the position is perfectly clear; to the practitioner the case for the anæsthetist needs no arguing. For no one is more cognisant of the responsibility that rests upon the administrator of the anæsthetic than is the general practitioner. Every medical man knows that there are occasions and cases when this responsibility is extraordinarily grave; yet few will hesitate to take it, to accept it to the full in the interests of their patients. Herein, in this calm acceptance of risk lies the heroism of the practitioner, a heroism of which the public knows little, because no one makes any fuss about it. So long as the practitioner (and the house surgeons at our hospitals stand in much the same position as does the general practitioner) decides to face this risk there will be "fatalities under anæsthetics," and no one acquainted with the facts would wish him to refuse to take his full share of responsibility, even in order to eliminate from coroners' courts such inquests as those which have occurred during the last few months.

There are many factors, as we have already pointed out, to be taken into consideration in discussing these unfortunate fatalities. A death under chloroform is very rarely a death from chloroform, though it is safe to say that the public regards it as such, and takes it, in fact, for granted that the patient died of an overdose of the anæsthetic. In none of the recent cases was there any reasonable evidence to show that the cause of death was solely due to the anæsthetic, and though it is, of course, impossible to eliminate the influence of the anæsthetising mixture used in every case, it is probable that in the majority of cases the special anæsthetic used had little to do with the fatal result. In other words, the patients would have succumbed, granted exactly similar conditions were present, no matter whether ether, chloroform, or A.C.E. had been used. This is an important point to bear in mind, and one not less important to remember in the discussion of the subject is the fact that the number of recent inquests does not show, as the public imagines it does, an exaggerated incompetency on the part of the officials of any particular institution. We hold no brief for any hospital in particular, but we venture to state that if an authoritative commission, able to gauge fully the relative importance of each factor, were to investigate the fatalities under anæsthetics at all our hospitals, the average figure for each hospital would almost exactly correspond. It must be borne in mind that it is difficult to define what is meant by the term death under anæsthetic. At some hospitals it is taken to mean that the patient has succumbed while unconscious; at others, while the patient is actually on the operating table. The former class of institution has consequently a higher mortality figure since its enumeration embraces cases which would be excluded from the latter, whose custom it is to hurry the patient off the table and on to the bed as soon as the slightest sign of danger becomes evident.

These are factors which it is not easy for the layman to grasp, or, having grasped, to weigh in order of their relative importance. But to do strict justice to the various institutions which have lately come before the public in an unfortunate light, owing to these inquests on deaths under anæsthetics, it is imperative that they should be grasped and properly weighed.

